# Exploring relationships between social media use, online exposure to drug-related content, and youth substance use in real time: a pilot ecological momentary assessment study in a clinical sample of adolescents and young adults

**DOI:** 10.3389/frcha.2024.1369810

**Published:** 2024-06-13

**Authors:** Meredith Gansner, Anna Katharine Horton, Rasika Singh, Zev Schuman-Olivier

**Affiliations:** ^1^ Department of Psychiatry, Cambridge Health Alliance, Cambridge, MA, United States; ^2^ Department of Psychiatry, Boston Children’s Hospital, Boston, MA, United States

**Keywords:** social media, adolescent, substance use, ecological momentary assessment, smartphone

## Abstract

**Introduction:**

Rising rates of adolescent overdose deaths attributed to counterfeit prescription drugs purchased using social media have drawn national attention to how these platforms might influence substance use. Research suggests a significant relationship exists between exposure to substance-related social media content and use of drugs and alcohol, but most studies are cross-sectional and limited by recall bias. This study used an ecological momentary assessment (EMA) protocol to collect longitudinal data on social media use and online drug-related exposures associated with youth substance use.

**Methods:**

Participants, aged 12–23, receiving mental health treatment from a U.S. community-based hospital, joined a six-week, smartphone-based EMA protocol. Each day, participants completed a modified CRAFFT screen for daily substance use and a survey on substance-related online content exposure, and input data from their smartphone screen time reports. Analyses employed mixed effects logistic regression models to explore relationships between substance-related online exposures, substance and social media use.

**Results:**

Data was obtained from 25 youth, predominantly white non-Hispanic/Latinx (56.0%) and female (64.0%). Participants had significantly higher odds of substance use on days when exposed to substance-related digital content posted by peers (OR: 19.6). They were also more likely to report these exposures (OR: 7.7) and use substances (OR: 29.6) on days when Snapchat was one of their most frequently used smartphone applications.

**Discussion:**

Our results support existing concerns about specific social media platforms being potential mediators of youth substance use. Future EMA studies in larger cohorts should explore the role of social media platforms in substance procurement.

## 1 Introduction

Adolescence is a complex developmental stage where youth experience significant social and neurocognitive changes, and it is common for individuals in this age group to initiate involvement in risk-taking behaviors such as substance use. Additionally, as the time teenagers spend on screens per day continues to rise, it is critical to understand how social media increases exposure to drug-related content and serves as a readily accessible route to obtain substances, which subsequently influences adolescent drug use (1, 2).

While regulations exist “prohibiting” drug and alcohol advertising to minors online, it is unclear how effective these regulations are; it is estimated that 53% of adolescents have seen Internet advertisements for cannabis (3). Furthermore, marketing teams have developed creative strategies to circumnavigate regulations. For example, e-cigarette product placement embedded within music videos has been connected to vaping in young adults (4), and these videos can be easily shared between teenage peers without detection on social media platforms.

Through these platforms, minors are also able to share their personal experiences with drugs and alcohol with their social media followers (5–7). Via popular video-sharing platforms like TikTok, youth encounter videos of online peers and fellow teenagers engaging in substance use or intoxication. This exposure not only strengthens the perception that substance use is prevalent among teenagers due to descriptive norms (8), but it also presents substance use in a favorable and entertaining light, aiming to capture the interest of a vast international audience (9–11). Of significant concern, social media platforms are also a means for youth to procure drugs (12–14). The increase in the percentage of fatal drug overdoses among adolescents in the U.S. has been attributed to counterfeit prescription pills contaminated with fentanyl; many of these overdoses have been anecdotally tied to pills purchased over social media platforms, and involve youth without formal substance use disorders (1, 2, 15).

Thus, just as the prevention of high-risk adolescent substance use requires recognition of real-world environmental influences, a detailed understanding of influences in the digital environment is likely just as critical. However, most information collected to-date about digital “risk factors” for adolescent substance use has been obtained through cross-sectional surveys. Mental health researchers have noted the limitations in using cross-sectional data to identify risk factors, as this approach is often incapable of capturing the dynamic and intricate interactions between participants and their environments (16, 17). Furthermore, adolescents are considered unreliable reporters of externalizing behaviors like substance use (18), and constant digital media engagment (19) may impede a teenager's ability to remember specific online exposures. The utilization of smartphone-based Ecological Momentary Assessment (EMA) enables researchers to capture the dynamics of the relationship between social media and substance use with improved granularity. Moreover, as researchers are increasingly recognizing the potential benefits of using smartphone based-interventions to address problematic adolescent substance use (20–22), findings from smartphone-based EMA studies can also inform the design of novel digital therapeutics that might intervene upon high-risk social media use associated with drug and alcohol use.

### 1.1 Current pilot study

In this pilot study, we experimented with the application of smartphone-based EMA, complemented by daily reports on smartphone screen time, to gather data on social media usage, online exposure to substance-related content, and substance use within a cohort of adolescents and young adults in outpatient mental health treatment. With the collected data, we aimed to identify temporal connections between use of the most popular social media platforms among youth in the United States, online exposure to drug-related content, and substance use.

Based on descriptive studies detailing diverse substance use-related content accessible through video-based platforms like YouTube and TikTok, we expected to uncover increased odds of exposure to drug-related content on days when participants reported frequent use of those platforms. Additionally, given cross-sectional research connecting substance use to viewing substance-related online content posted by one's peers (6, 8), we anticipated to see more instances of substance use on days when participants' peers shared drug or alcohol-related content online.

## 2 Methods

### 2.1 Recruitment and study procedure

This longitudinal cohort study investigated the social media exposures and substance use behaviors of a group composed of individuals aged 12–23 (*N* = 25) from outpatient psychiatry clinics within a community-based hospital system over a four-month duration, spanning from October 2021 to January 2022. The recruitment site was chosen both because of the known correlation between negative affective symptoms and risk of youth substance use (23), and in order to leverage the existing recruitment procedures already in place for similar EMA studies within the hospital system.

### 2.2 Participants

Youth could take part in the study by providing their consent or assent, with guardians offering informed consent for those under 18. Eligibility criteria included owning a personal Android or iPhone and having the ability to read English at a 6th-grade level. Participants were not required to have a history of substance use in order to participate in the study. Recruitment efforts involved distributing clinic fliers, receiving referrals from clinicians, and reaching out to eligible clinic patients through emails and phone calls. Prospective participants, along with guardians if applicable, had an initial meeting with a study team member to complete the consent/assent process and receive instructions on using the study's Ecological Momentary Assessment (EMA) application. Demographic data including age, gender and race/ethnicity were collected from the participant's electronic medical record at the start of the study period. Each participant's psychiatric diagnoses were also collected from the electronic medical record at this time.

### 2.3 Daily survey and data collection

Participants used mindLAMP (24), a non-commercial, smartphone-based digital phenotyping application, to complete nightly questionnaires for six weeks. Questionnaires included an item on daily substance use (Part A of the CRAFFT screening tool) (25), anxiety symptoms (GAD-7) (26), and depression symptoms (PHQ-8) (27). The CRAFFT screening tool is a two-part validated screening instrument used to assess drug and alcohol use in youth 12–21 years of age; part A of the tool includes three questions pertaining to alcohol use, marijuana use, and use of illegal drugs (25). The GAD-7 and PHQ-8 questionnaires assessed the severity of participants' internalizing symptoms (anxiety via the 7-item GAD-7 and depression via the 8-item PHQ-8). All screening tools were modified for daily use in order to obtain as specific a relationship between variables of interest as possible. For example, where the original GAD-7 asks “Over the last two weeks, how often have you been bothered by feeling nervous, anxious, or on edge”, our instrument asked participants to reflect only on their symptoms only in the last 24 h, with answer choices also modified accordingly from “several days” to “several times”, “more than half the days” to “more than half of the day”, and “nearly every day” to “nearly all day”. Participants were also asked yes/no questions about exposures to drug-related content online that day (any exposure, intentional, and peer-mediated). They were not asked to describe the exact nature of an exposure (e.g., video or image, which platform), only whether such an exposure was encountered. An “intentional exposure” was any drug or alcohol-related online content that had intentionally been sought out by the participant on that day. A “peer-mediated” exposure referred to any drug or alcohol-related content that had been shared or posted online by someone that participant considered to be a peer. A peer did not specifically have to be someone that the participant knew “in-person”. At the end of the daily survey, participants were asked to look at their daily iPhone or Android screen time reports and input the three apps or websites they spent the most time using that day.

Study participants were sent nightly reminders to fill-out EMA questionnaires via a single push notification at 8:00 in the evening. Youth were compensated for their participation with a $50 Amazon gift card provided at the end of the study period. Full compensation was given to participants independent of the number of surveys completed. The study's protocol was approved by the hospital system's institutional review board.

### 2.4 Data processing and analysis

Raw data was processed using Excel. Participants with no completed surveys were removed from analyses. Each daily entry was coded 1 or 0 for each of the four most popular social media platforms among U.S. adolescents (i.e., YouTube, TikTok, Instagram and Snapchat). For example, on a day when both TikTok and Instagram were included in a participant's three most frequently used smartphone applications, a 1 would be coded for both platforms. Other social media platforms were to be included in the analyses if they were used by more than five participants during the study.

Mixed-effects logistic regression models were constructed to determine associations between types of digital media use, exposures to drug-related content, and instances of substance use. Primary analyses were controlled for age, gender, race/ethnicity, study time point, and active symptoms of anxiety and depression (via GAD-7 and PHQ-8 scores respectively). Statistical precision was determined with 95% confidence intervals. Analyses were performed using Stata v.14.2. Analyses were not performed for variables where there was insufficient data (e.g., no instances of a specific type of digital media use on days when substance use occurred). To account for multiple comparisons, the Benjamini-Hochberg procedure was implemented with a false discovery rate (FDR) of 0.15, based on FDR recommendations for pilot studies (28).

## 3 Results

Our study recruited 28 youth (*N* = 28) to participate in the study, and 25 youth (*n* = 25) provided the requisite data for analyses. The three excluded participants provided insufficient data regarding outcomes of interest. Of the 25 participants with sufficient data, the average age was 16.0 years, ranging from 12 to 19 years of age. Participants primarily identified as white, non-Hispanic/Latinx (14/25: 56.0%), followed by Black and biracial (both 4/25: 16.0%), and then Hispanic/Latinx (3/25: 12.0%). The majority of participants identified as cisgender female (16/25: 64.0%), with 28.0% (7/25) identifying as cisgender male, and 8.0% (2/25) as non-binary. Study participants had primarily been diagnosed with depressive disorders, anxiety disorders, trauma-related disorders and Attention-Deficit/Hyperactivity Disorder (ADHD); no participant had been diagnosed with a primary substance use disorder. Complete information regarding participant demographics and psychiatric diagnoses can be found in Table 1.

**Table 1:** Participant demographics and diagnoses.

Gender	Male	Female	Non-binary
Overall: *n* (%)	7	16	2
Age (Mean)	16	15.9	16
Race/Ethnicity: *n* (%)			
White, non-Hispanic/Latinx	4	9	1
Black	0	3	1
Hispanic/Latinx	1	2	0
Asian	0	0	0
Biracial	2	2	0
Diagnostic categories^a^			
Depressive disorder	7	13	1
Anxiety disorder	5	14	0
ADHD	4	2	0
Trauma-related disorder	0	2	1
Eating disorder	0	1	1
Psychotic disorder	0	1	0
Adjustment disorder	0	0	1
Substance use disorder	0	0	0

^a^ Multiple participants had more than one primary diagnosis.

Complete data was captured for approximately 34% of the total number of days captured across the total sample (42 days for 25 participants). As participants were not compensated based on the number of surveys completed, participant dropout increased as the study period progressed; of the 25 participants, 18 (72%) continued to engage with the app by their third week of the study, and 10 participants (40%) continued to engage with the app by their fifth week.

All 25 participants reported one of the four most popular social media platforms as a top used app on at least one day during their study period. Instagram was the platform cited as a top app by the most participants (19/25: 76.0%), followed by TikTok (16/25: 64.0%), Snapchat (15/25: 60.0%), and then YouTube (13/25: 52.0%). However, TikTok was reported as a top app most often, on 42.6% of the total response days, followed by YouTube (40.9%), Instagram (35.7%), and then Snapchat (20.0%). The majority of response days (87.5%) included at least one of these four social media platforms as a top used app, No other social media platforms were used by more than five of the study's participants.

Of the 25 participants, 18 (72.0%) reported a drug-related online exposure during the study, while 15 (60.0%) reported seeing drug-related content posted online by their peers, and 7 (28.0%) reported intentionally seeking out drug-related online content. Out of the total response days from the entire cohort, drug-related online exposure was reported on 29.6% of days, peer-mediated online exposure on 13.9% of days, and intentional drug-related exposures on 2.9% of total days.

Six participants reported using substances during the study period; the majority identified as cisgender female and half as white, non-Hispanic/Latinx). The average age of youth who reported substance use during the study was 16.3. Across these six participants, eight instances of substance use were reported, primarily involving cannabis (6/8: 75%). Alcohol use was reported once (1/8: 12.5%), and use of an unnamed illicit drug was reported once as well (1/8: 12.5%).

Episodes of substance use were more likely to be reported on days when participants' peers had posted drug-related content online (OR: 19.6, *p* = .04); they were not more likely to occur on days when the participants themselves intentionally sought out or posted drug-related digital content (Table 2). Additionally, participants had significantly higher odds of encountering that peer-posted, drug-related online content when Snapchat and TikTok were among the top used smartphone applications that day, but lower odds when YouTube was listed as a top used app (Table 3). Frequent Snapchat use in particular appeared to confer the greatest risk of peer-mediated online exposures (Snapchat OR: 7.66, *p* = .001 vs. TikTok OR: 4.02, *p* = .023). Instances of substance use were also significantly more likely to occur on days when Snapchat was a top-used application (OR: 29.6, *p* = .03) (Table 4).

**Table 2:** Relationships between substance use and online drug-related exposures^a^.

Category of online exposure	OR	95% CI		*p* ^b^
		Lower	Upper	
Any exposure	5.37	0.38	75.6	.21
Intentional exposure	6.10	0.82	45.7	.08
Peer-mediated exposure	19.6	1.22	314.8	*.04

^a^ Analyses controlled for age, gender, race, study timepoint, and daily depressive/anxiety symptoms.

^b^ *denotes significance following Benjamini-Hochberg procedure.

**Table 3:** Relationships between social media use and online drug-related exposures^a^.

	Category of online exposure	OR	95% CI		*p* ^b^
			Lower	Upper	
Any exposure					
Social media platform listed as “Top Used App” that day^c^	Snapchat	3.20	1.16	8.88	*.03
	TikTok	2.00	0.84	4.74	.12
	Instagram	0.87	0.43	1.77	.70
	YouTube	0.76	0.32	1.79	.53
Intentional exposure					
Social media platform listed as “Top Used App” that day^c^	Snapchat	2.89	0.48	17.6	.25
	TikTok	2.66	0.30	23.9	.38
	Instagram	3.09	0.58	16.4	.19
	YouTube	2.87	0.36	23.0	.32
Peer-mediated exposure					
Social media platform listed as “Top Used App” that day^c^	Snapchat	7.66	2.32	25.3	*.001
	TikTok	4.02	1.17	13.8	*.03
	Instagram	1.17	0.46	2.98	.75
	YouTube	0.17	0.05	0.64	*.01

^a^ Analyses controlled for age, gender, race, study timepoint, and daily depressive/anxiety symptoms.

^b^ * denotes significance following Benjamini-Hochberg procedure.

^c^ Compared to days when platform not listed as top used app.

**Table 4:** Relationships between type of digital media use and substance use events^a^.

		OR	95% CI		*p* ^b^
			Lower	Upper	
Social media platform listed as “Top Used App” that day^c^	Snapchat	29.6	1.40	626.0	*.03
	TikTok	2.28	0.08	63.3	.63
	Instagram	0.10	0.001	18.2	.39
	YouTube	1.59	0.14	18.1	.71

^a^ Analyses controlled for age, gender, race, study timepoint, and daily depressive/anxiety symptoms.

^b^ * denotes significance after correction using Benjamini-Hochberg procedure.

^c^ Compared to days when platform not listed as top used app.

In post-hoc analyses, multivariate mixed effects models including all four social media platforms were constructed in order to examine relationships of interest. Snapchat remained significantly associated with both instances of substance use and drug-related online exposures (overall and peer-mediated), while YouTube was associated with higher odds of intentionally searching for drug-related digital content (Supplementary Table S1). Multicollinearity in social media platform use was assessed by examination of tolerance (cutoff <0.1) and variance inflation factors (cutoff >5.0); variables didn't exceed acceptable limits.

## 4 Discussion

To our knowledge, this is the first study to pilot use of smartphone-based EMA to examine the temporal relationships between discrete instances of youth substance use, drug-related online exposure, and use of specific social media platforms, and our findings may have important implications for the study of high-risk youth substance use.

Our finding that participants had higher odds of substance use on days when they saw their peers posting about drugs and alcohol online further supports the significance of peer influence on youth substance use. Prior cross-sectional research performed by our study team similarly identified more severe substance use in youth whose peers posted drug-related content, even if the participant themselves didn't post such online content.^1^ Whether or not visualizing content of one's peers using substances is more influential than visualization of other drug-related content remains an outstanding question. Certainly, with substances like cannabis and alcohol, it is not uncommon for youth to encounter social media content of a peer intoxicated or engaging in substance use (29).

In both bivariate and multivariate analyses incorporating all social media platforms, frequent TikTok and Snapchat use were uniquely associated with higher odds of peer-mediated exposure to drug-related digital content. As a direct messaging app, Snapchat's ephemeral texting feature makes it an ideal platform for peers to share drug-related content privately with one another. TikTok's current status as the predominant video-sharing platform likely explains why participants were more likely to encounter peer-mediated drug-related content on days when they used the app more frequently. Conversely, decreased odds of peer-mediated drug-related content exposure on days when YouTube was a top-used app may suggest that YouTube is no longer the platform for video sharing among one's peers. Rather, YouTube's association with intentional drug-related exposure in our post-hoc analyses may highlight the nature of drug-related content the participant was seeking. Youth seeking comprehensive videos about drug or alcohol use may choose to seek out more diverse content (in quality and quantity) on YouTube, where videos can exceed TikTok's time limits.

Our results also support existing concerns about Snapchat as a potential mediator of youth substance use (2, 30). Our study did not ask participants how they had obtained drugs or alcohol, but social media platforms are increasingly being used to facilitate drug procurement (13, 29), and Snapchat in particular has attracted national attention due to anecdotal reports suggesting that drug dealers might be targeting buyers, including minors, via the platform (1, 2). Considering that fentanyl contamination can make a single instance of prescription drug misuse lethal, potentially easy and clandestine access for adolescents to any drug via social media is a cause for concern. The clinical implications of these results, and the results of future studies in this area are notable; practitioners who treat youth with problematic substance use may choose to incorporate social media platform-specific screening in their practice, and focus on reducing youth engagement with higher-risk platforms (e.g., turn off platform notifications). Ecological momentary intervention protocols could also be designed to identify and flag a youth's drug-related social media use in real time, augmenting existing substance use treatment.

Lastly, while adjusting for co-occurring anxiety and depression symptoms did not appear to alter our significant findings, there remains uncertainty regarding aspects of this undoubtedly complex relationship. For example, transient worsening in affective symptoms might have a differing impact on the social media habits or engagement with drug-related online content in youth without existing psychiatric diagnoses. Furthermore, both the Internet and drugs and alcohol are used by adolescents and young adults to cope with negative emotions (31, 32), such that it would not be unexpected for higher internalizing symptoms to moderate a potential relationship between exposure to drug-related online content and substance procurement via social media platforms. As such, future studies on this topic should also measure affective symptoms to determine the exact nature of their influence.

## 5 Limitations

Our pilot study has several limitations, most of which we plan to address in larger well-powered EMA studies based on this initial protocol. Our sample size is small, a clinical population, and representative of only a localized geographical region within a single U.S. state. We also captured a relatively few number of instances of substance use, and did not obtain objective assessments of participant drug use, which makes our findings of a significant relationship between Snapchat use and substance use one that should be interpreted with caution, and ideally viewed as a relationship needing further exploration.

Concerning these specific limitations, even with sample sizes under 50, studies collecting repeated, longitudinal measurements may still be adequately powered to detect clinically meaningful changes in symptoms or behaviors (33, 34). Furthermore, use of these four social media platforms is pervasive among youth both in the United States and internationally, such that our initial findings might still have widespread relevance, even in non-clinical populations. However, larger well-powered studies that incorporate objective assessment of participant substance use would increase the odds of capturing more substance use events, and help to clarify how social media use might be temporally related to substance procurement and specific types of drugs or alcohol use with greater certainty.

## 6 Conclusion

Despite widespread use of social media by youth, questions remain regarding how these platforms influence high-risk adolescent behaviors, like substance use. Smartphone-based ecological momentary assessment that incorporates passive sensor data collection can improve our understanding of the relationship between an adolescent's online experiences and their use of drugs and alcohol. Using this methodology, our study offers novel evidence that specific social media platforms and content may be more relevant for mental health clinicians to consider when addressing adolescent substance use. Clinicians should offer guidance surrounding avoidance of high-risk social media platforms to those adolescent patients who turn impulsively to recreational drug use to manage internalizing symptoms. Lawmakers might choose to hold social media platforms legally accountable for the ease with which drug-related content is uploaded and shared among juvenile platform users. Finally, additional longitudinal studies combining EMA and passive sensor data collection should focus on identifying the specific types of peer-posted online content most connected to subsequent substance procurement and use in youth.

## Data Availability

The original contributions presented in the study are included in the article/**Supplementary Materials**, further inquiries can be directed to the corresponding author.

## Footnotes

^1^Gansner M, Nisenson M, Si R, Singh R, Marsch LA, Schumann-Olivier Z. Understanding the relationship between online exposure to drug-related content, digital Media use, and drug use in a sample of U.S. youth. (2023).
