# Supplementary material for: Exploring relationships between social media use, online exposure to drug-related content, and youth substance use in real time: a pilot ecological momentary assessment study in a clinical sample of adolescents and young adults
Source: Front Child Adolesc Psychiatry. 2024 Jun 13;3:1369810. doi: 10.3389/frcha.2024.1369810 (PMC11745144; doi:10.3389/frcha.2024.1369810)
Supplement: Supplementary file 1 [file Table1.docx]

| **SUPPLEMENTAL TABLE 1: POST-HOC ANALYSES BETWEEN TYPE OF SOCIAL MEDIA PLATFORM, SUBSTANCE USE, AND ONLINE DRUG-RELATED EXPOSURES** | | | | |  |
| --- | --- | --- | --- | --- | --- |
|  | **Social Media Platform Subtype** | **OR** | **95% CI** | | **p^b^** |
|  |  |  | *lower* | *upper* |  |
| **Any Drug-Related Exposure** | *Snapchat* | 3.61 | 1.28 | 10.2 | *.02 |
|  | *TikTok* | 2.28 | 0.94 | 5.55 | .07 |
|  | *Instagram* | 0.83 | 0.40 | 1.73 | .61 |
|  | *YouTube* | 1.03 | 0.42 | 2.52 | .96 |
| **Intentional Exposure** | *Snapchat* | 5.51 | 0.97 | 31.4 | .06 |
|  | *TikTok* | 7.59 | 0.76 | 75.9 | .08 |
|  | *Instagram* | 5.08 | 0.86 | 30.0 | .07 |
|  | *YouTube* | 14.6 | 1.31 | 162.3 | *.03 |
| **Peer-mediated Exposure** | *Snapchat* | 6.99 | 2.22 | 22.0 | *.001 |
|  | *TikTok* | 4.38 | 1.34 | 14.4 | *.02 |
|  | *Instagram* | 1.04 | 0.40 | 2.74 | .93 |
|  | *YouTube* | 0.36 | 0.09 | 1.40 | .14 |
| **Substance Use** | *Snapchat* | 36.3 | 2.06 | 637.5 | *.01 |
|  | *TikTok* | 1.45 | 0.15 | 14.1 | .75 |
|  | *Instagram* | 0.24 | 0.02 | 2.85 | .26 |
|  | *YouTube* | 5.49 | 0.38 | 78.9 | .21 |
| ^a^ Analyses controlled for age, gender, race and study timepoint;  ^b^ * denotes significance after correction via Benjamini-Hochberg procedure | | | | |  |
